# miR-373-3p Targets DKK1 to Promote EMT-Induced Metastasis via the Wnt/**β**-Catenin Pathway in Tongue Squamous Cell Carcinoma

**DOI:** 10.1155/2017/6010926

**Published:** 2017-02-27

**Authors:** Junquan Weng, Hui Zhang, Cheng Wang, Jianfeng Liang, Guanhui Chen, Wenqing Li, Haikuo Tang, Jinsong Hou

**Affiliations:** ^1^Department of Oral and Maxillofacial Surgery, Hospital of Stomatology, Guanghua School of Stomatology, Sun Yat-sen University, Guangzhou, Guangdong 510055, China; ^2^Department of Stomatology, Shenzhen People's Hospital, Second Clinical Medical School of Jinan University, Shenzhen, Guangdong 518020, China; ^3^Department of Stomatology, Pingshan New District People's Hospital of Shenzhen, Shenzhen, Guangdong 518118, China; ^4^Guangdong Key Laboratory of Stomatology, Sun Yat-sen University, Guangzhou, Guangdong 510080, China

## Abstract

MicroRNAs (miRNAs) regulate gene expression and at the same time mediate tumorigenesis. miR-373-3p has diverse effects in tumors, but its role in tongue squamous cell carcinoma (TSCC) remains unknown. The purpose of this study is to determine the function of miR-373-3p in the progression of TSCC. Our results brought to light that miR-373-3p is markedly upregulated in clinical TSCC tissues compared with paired adjacent normal tissues and has significant correlation with a more aggressive TSCC phenotype in patients. Gain-of-function and loss-of-function studies revealed that ectopic miR-373-3p overexpression promoted the metastasis of TSCC cells. Notably, Wnt/*β*-catenin signaling was hyperactivated in TSCC cells overexpressing miR-373-3p, and this pathway was responsible for the epithelial-mesenchymal transition (EMT) induced by miR-373-3p. Furthermore, miR-373-3p directly targeted and suppressed Dickkopf-1 (DKK1), a negative regulator of the Wnt/*β*-catenin signaling cascade. These results demonstrate that, by directly targeting DKK1, miR-373-3p constitutively activated Wnt/*β*-catenin signaling, thus promoting the EMT-induced metastasis of TSCC. Taken together, our findings reveal a new regulatory mechanism for miR-373-3p and suggest that miR-373-3p might be a potential target in TSCC therapy.

## 1. Introduction

Tongue squamous cell carcinoma (TSCC) is the most common cancer of the oral cavity and is a very aggressive disease [1]. Over the past several decades the survival rate for patients with TSCC has not significantly improved despite the progress in treatment. Local or regional relapse and cervical lymph node metastasis are considered to be the most prevalent causes of death in these patients [2]. It was revealed by several researches that epithelial-mesenchymal transition (EMT) plays a significant role during TSCC metastasis and progression [3, 4]. During EMT, epithelial cells acquire a spindle-shaped, fibroblastic-like phenotype and mesenchymal properties while losing epithelial polarity, collectively leading to enhanced cell motility and invasiveness [5].

The Wnt/*β*-catenin signaling pathway is an established regulator of the EMT due to its involvement in the maintenance of epithelial integrity and tight adherens junctions [6]. Mutations of the proteins in this pathway often contribute to TSCC metastasis and progression [7]. Upon Wnt pathway activation, *β*-catenin is stabilized and allowed to accumulate. Activated *β*-catenin dissociates from E-cadherin, which disassembles adherens junctions and then enters the nucleus to activate the expression of target genes, which accounts for the upregulation of EMT biomarkers and an ultimately unfavorable cancer prognosis [6, 8]. Dickkopf-1 (DKK1), a transcriptional target of the Wnt/*β*-catenin pathway, functions as a negative regulator of Wnt signaling [9]. It has been reported that Wnt signaling inhibition by DKK1 limits the invasiveness of various types of cancer and that pathway activation by lithium chloride normally promotes cancer cell metastasis [9–11]. Accordingly, the Wnt/*β*-catenin signaling pathway is considered an important regulator of cancer cell metastasis; therefore, more in-depth researches of the mechanisms that regulate the Wnt/*β*-catenin pathway may provide a new perspective for more effective TSCC therapies.

miRNAs are a class of small noncoding RNAs that are approximately 22 nucleotides in length. Typically, miRNAs bind to the complementary sequence in the 3′-untranslated regions (3′-UTRs) of mRNAs, leading to the degradation or inhibited translation of the targeted mRNAs [12]. Bioinformatic predictions suggest that miRNAs potentially regulate more than 30% of the protein-coding genes [12]. These highly conserved miRNAs have been implicated in the regulation of many factors contributing to TSCC progression, including growth and metastasis, which means that they can function either as tumor suppressors or oncogenes [13–15]. For instance, miR-133a and miR-133b, which inhibit cell proliferation by targeting pyruvate kinase type M2, are frequently downregulated in TSCC [14]. miR-21 is significantly upregulated in highly metastatic TSCC cell lines and metastatic tissues, and the level of miR-21 is inversely correlated with tropomyosin 1 (TPM1) and phosphatase and tensin homologue deleted on chromosome ten (PTEN) expression and TSCC cell apoptosis [15]. These studies suggest important links between miRNAs and the development, progression, metastasis, and prognosis of TSCC. Previous studies have also shown that miR-373-3p functions in multiple cellular processes and that its deregulation is a hallmark of human cancer [16–18]. However, a detailed understanding of the expression and mechanistic function of miR-373-3p in TSCC remains unclear.

In this study, we sought to elucidate the effect of miR-373-3p on Wnt/*β*-catenin signaling in TSCC and to discern the relevant molecular mechanisms. Our data show that miR-373-3p is upregulated in tissues isolated from patients with TSCC. Furthermore, miR-373-3p activates the Wnt/*β*-catenin pathway by directly targeting DKK1 to consequently promote EMT and metastasis of TSCC.

## 2. Materials and Methods

### 2.1. Tissues and Cells

Fresh TSCC tissue samples from TSCC patients and their adjacent nonneoplastic tissue samples were collected with informed consent under the institutional board-approved protocols from the Hospital of Stomatology, Sun Yat-sen University (Guangzhou, China). The samples had been clinically and histopathologically diagnosed according to the World Health Organization criteria. Tumor and noncancerous tissues were confirmed histologically by hematoxylin and eosin staining. This study was approved by the institutional research ethics committee of the Hospital of Stomatology, Sun Yat-sen University. TSCC cell lines SCC-9, SCC-15, SCC-25, UM1, and UM2 were routinely maintained in DMEM (Invitrogen, Carlsbad, CA) supplemented with 10% fetal bovine serum (HyClone, Logan, UT).

### 2.2. Constructs, Reagents, and Assays

The wild-type 3′-UTR of human DKK1 was subcloned into pmirGLO using SpeI and HindIII cut sites (Promega, Madison, WI, USA). Mutations of the 3′-UTR sequence were created with a Quick Change Site-Directed Mutagenesis Kit (SBS Genetech, Beijing, China). HEK293T cells were seeded onto 24-well plates (1 × 10^5^ cells per well) the day before transfection. Cells were then transfected with the firefly luciferase reporter plasmid including either the wild-type or mutant 3′-UTR of DKK1 (50 ng per well) and the pRL-TK* Renilla* luciferase reporter (10 ng per well). The cells were then transfected with miR-373-3p mimics or antagomiR-373-3p (50 nM) using Lipofectamine 2000 (Invitrogen). Luciferase and* Renilla* signals were measured 24 h after transfection using a Dual Luciferase Reporter Assay Kit (Promega).

### 2.3. Oligonucleotides and Transient Transfection

miR-373-3p mimics, antagomiR-373-3p, siRNA-DKK1 (siDKK1), siRNA-*β*-catenin (si*β*-catenin), siRNA-T cell factor 4 (siTCF4), and corresponding control oligonucleotides (RiboBio, Guangzhou, China) were transfected into SCC-9 and UM1 cells using Lipofectamine 2000 (Invitrogen) according to the manufacturer's instructions. The sense strand sequences of siRNAs were as follows: siDKK1, 5′-GAAUAUGUGUGUCUUCUGAUU-3′; si*β*-catenin, 5′-UGGUUGCCUUGCUCAACAA-3′; and siTCF4, 5′-AAGUCCGAGAAAGGAAUCUGA-3′.

### 2.4. RNA Extraction, Reverse Transcription, and Real-Time RT-PCR

Total miRNA from cultured cells and fresh surgical tongue tissues was isolated using TRIzol (Invitrogen). Detection of miR-373-3p and mRNA was performed as previously described [15]. *β*-Actin and U6 were used to normalize mRNA and miRNA, respectively. The sequences of the primers were as follows: E-cadherin forward, 5′-GACCGGTGCAATCTTCAAA-3′; E-cadherin reverse, 5′-TTGACGCCGAGAGCTACAC-3′; vimentin forward, 5′-TTCTTCCCTGAACCTGAGAGA-3′; vimentin reverse, 5′-GAGTGGGTGTCAACCAGAGG-3′; N-cadherin forward, 5′-GTGCCATfAGCCAAGGGAA-3′; N-cadherin reverse, 5′-GCGTTCCTGTTCCACTCATAGGAG-3′; CK18 forward, 5′-AAGGCCTACAAGCCCAGATT-3′; CK18 reverse, 5′-CACTGTGGTGCTCTCCTCAA-3′; CCND1 forward, 5′-TTCTGCCTTTGATGTTAC-3′; CCND1 reverse, 5′-AGGCTGAATCAATGTCTT-3′; c-Myc forward, 5′-TCAAGAGGCGAACACACAAC-3′; c-Myc reverse, 5′-GGCCTTTTCATTGTTTTCCA-3′; LEF1 forward, 5′-CACTGTAAGTGATGAGGGGG-3′; LEF1 reverse, 5′-TGGATCTCTTTCTCCACCCA-3′; MMP-7 forward, 5′-GCATCTCCTTGAGTTTGGCT-3′; MMP-7 reverse, 5′-GAGCTACAGTGGGAACAGGC-3′; GAPDH forward, 5′-GACTCATGACCACAGTCCATGC-3′; and GAPDH reverse, 5′-AGAGGCAGGGATGATGTTCTG-3′. Relative quantitation was calculated using 2^−ΔΔCt^ method. All experiments were carried out in triplicate.

### 2.5. Cell Viability Analysis

CCK-8 assay was performed to detect cell viability according to a standard method, as previously described [19].

### 2.6. Cell Invasion Assay

The invasion assay was performed using a transwell chamber consisting of 8 mm membrane filter inserts (Costar, Corning, NY, USA). Briefly, the upper chamber was coated with Matrigel (BD Biosciences, Franklin Lakes, NJ, USA) and incubated for 24 h. The noninvading cells were gently removed with a soft cotton swab, and the cells that invaded to the bottom chamber were fixed with 4% paraformaldehyde, stained with hematoxylin, photographed, and counted.

### 2.7. Western Blot Analysis

Western blotting was performed according to a standard method, as previously described [19]. The following primary antibodies were used: anti-*β*-catenin and anti-E-cadherin (1 : 500, Cell Signaling Technology, Danvers, MA, USA); anti-DKK1, anti-cytokeratin 18, anti-N-cadherin, and anti-vimentin (1 : 1000, Cell Signaling Technology); Anti-Nuclear Matrix Protein p84 (1 : 500, Abcam, Cambridge, UK); and anti-*α*-GAPDH and anti-*α*-tubulin antibody (1 : 1000, Sigma-Aldrich, St. Louis, MO, USA). Nuclear extracts were prepared using the Nuclear Extraction Kit (Active Motif, Carlsbad, CA, USA).

### 2.8. Luciferase Assays

Cells (4 × 10^4^) were seeded in triplicate onto 24-well plates and cultured for 24 h. Cells were transfected with 100 ng TOP/FOP reporter luciferase plasmid, pmirGLO-DKK1-3′-UTR, or pmirGLO-DKK1-3′-UTR-mut luciferase plasmids, each in combination with 5 ng of the pRL-TK* Renilla* plasmid (Promega) using Lipofectamine 2000 (Invitrogen) according to the manufacturer's recommendation. Luciferase and* Renilla* signals were measured twenty-four hours after transfection using a Dual Luciferase Reporter Assay Kit (Promega) according to the manufacturer's protocol.

### 2.9. Bioinformatics Analysis

The following online software programs were used for bioinformatics analysis: TargetScan 4.1 (http://targetscan.org/vert_40/); DIANA-mirPath (http://diana.imis.athena-innovation.gr/); and miRanda (http://www.microrna.org/microrna/getGeneForm.do).

### 2.10. Statistics

All statistical analyses were performed using SPSS 18.0 statistical software (SPSS Inc., Chicago, IL, USA). Statistical differences were determined by the two-tailed Student's *t*-test between two groups of data; a *P* value < 0.05 was considered statistically significant.

## 3. Results

### 3.1. miR-373-3p Is Upregulated in Human TSCC Tissues and Cell Lines

**Figure 1:**
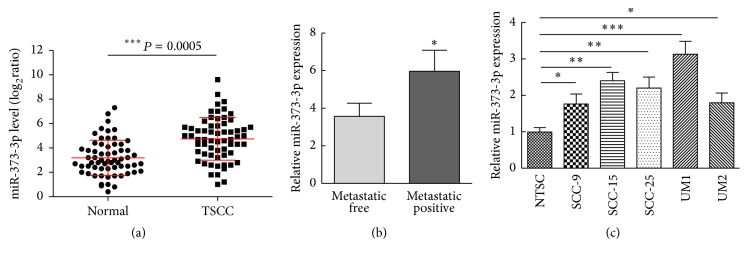
miR-373-3p is upregulated in TSCC tissues and correlates with metastatic capacity. (a) Expression of miR-373-3p in an independent validation cohort of 63 pairs of matching normal and TSCC tissues. (b) Mean values of miR-373-3p relative levels from TSCC tissues including a group of 46 TSCC patients with positive lymph node metastases compared to the control group of 15 TSCC patients with negative lymph node metastases. (c) Expression levels of miR-373-3p in normal tongue squamous cells (NTSCs) and five TSCC cell lines (SCC-9, SCC-15, SCC-25, UM1, and UM2). All assays were performed in duplicate. ^*∗*^*P* < 0.05, ^*∗∗*^*P* < 0.01, and ^*∗∗∗*^*P* < 0.001.

To identify the role of miR-373-3p in the development of TSCC, we analyzed the expression of miR-373-3p by quantitative real-time PCR (qRT-PCR) in 63 pairs of TSCC and matched control adjacent normal tissues isolated from patients. Compared to normal tissues, the average expression level of miR-373-3p was significantly increased in tumor tissues (Figure 1(a)). Further statistical analysis was performed to assess the clinicopathological significance of miR-373-3p expression in the 63 patients with TSCC, and it turned out that higher miR-373-3p expression was observed in primary tumors that subsequently metastasized than in those that did not metastasize (Figure 1(b)). We then assessed the expression of miR-373-3p in normal tongue squamous cells (NTSCs) and five TSCC cell lines (SCC-9, SCC-15, SCC-25, UM1, and UM2) and found that miR-373-3p was upregulated in TSCC cells compared with NTSCs (Figure 1(c)). Our results suggest that miR-373-3p is upregulated in TSCC, and the upregulation of miR-373-3p is correlated with TSCC progression, which suggests a metastasis-promoting function of miR-373-3p in TSCC.

### 3.2. miR-373-3p Promotes TSCC Cell EMT and Invasion In Vitro

**Figure 2:**
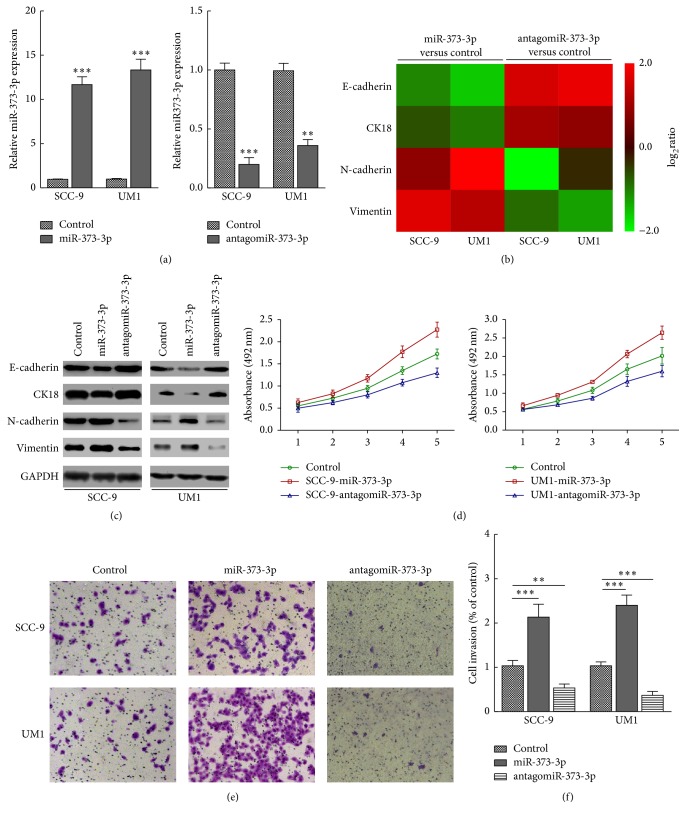
miR-373-3p promotes TSCC cell EMT and invasion in vitro. (a) After SCC-9 and UM1 cells were transfected with miR-NC (50 nM), miR-373-3p (50 nM), and orantagomiR-373-3p (50 nM) for 24 h, the efficacy of miR-373-3p expression was determined by qRT-PCR. ((b) and (c)) Real-time PCR (b) and Western blotting (c) analysis revealed that miR-373-3p regulates the expression levels of numerous EMT regulators. The pseudocolors represent the log_2_ transformed intensity scales of miR-373-3p expression versus control-transfected cells or antagomiR-373-3p versus control-transfected cells. (d) Proliferation curves showing the effect of miR-373-3p on cell proliferation. ((e) and (f)) Representative micrographs and histograms depicting the invasion of SCC-9 and UM1 cells after miR-NC (50 nM), miR-373-3p (50 nM), or antagomiR-373-3p (50 nM) transfection. The data are presented as the mean ± s.d. of three independent experiments. ^*∗∗*^*P* < 0.01, and ^*∗∗∗*^*P* < 0.001.

By transfecting miR-373-3p mimics into the human TSCC cell lines SCC-9 and UM1, in vitro gain-of-function analyses were performed to explore the effects of miR-373-3p deregulation on the invasiveness of TSCC cells (Figure 2(a)). As shown in Figures 2(b) and 2(c), the mRNA and protein levels of epithelial markers, including E-cadherin and CK18, were drastically downregulated. On the contrary, mesenchymal markers such as vimentin and N-cadherin were dramatically upregulated in miR-373-3p-transfected SCC-9 and UM1 cells. These results suggest that miR-373-3p might promote transition from an epithelial to a mesenchymal phenotype. Consistent with this hypothesis, CCK-8 and Matrigel-coated transwell assays showed that miR-373-3p overexpression drastically enhanced the viability (Figure 2(d)) and invasiveness (Figures 2(e) and 2(f)) of SCC-9 and UM1 TSCC cells.

To further investigate the proinvasive role of miR-373-3p in TSCC, we examined the effect of inhibiting miR-373-3p on the mesenchymal phenotype of SCC-9 and UM1 cells. As expected, miR-373-3p inhibition markedly decreased the EMT (Figures 2(b) and 2(c)) and the viability (Figure 2(d)) and invasiveness of both SCC-9 and UM1 cells (Figures 2(e) and 2(f)). Collectively, our data suggest that miR-373-3p contributes significantly to the metastasis of TSCC.

### 3.3. miR-373-3p Activates the Wnt/*β*-Catenin Signaling Pathway

**Figure 3:**
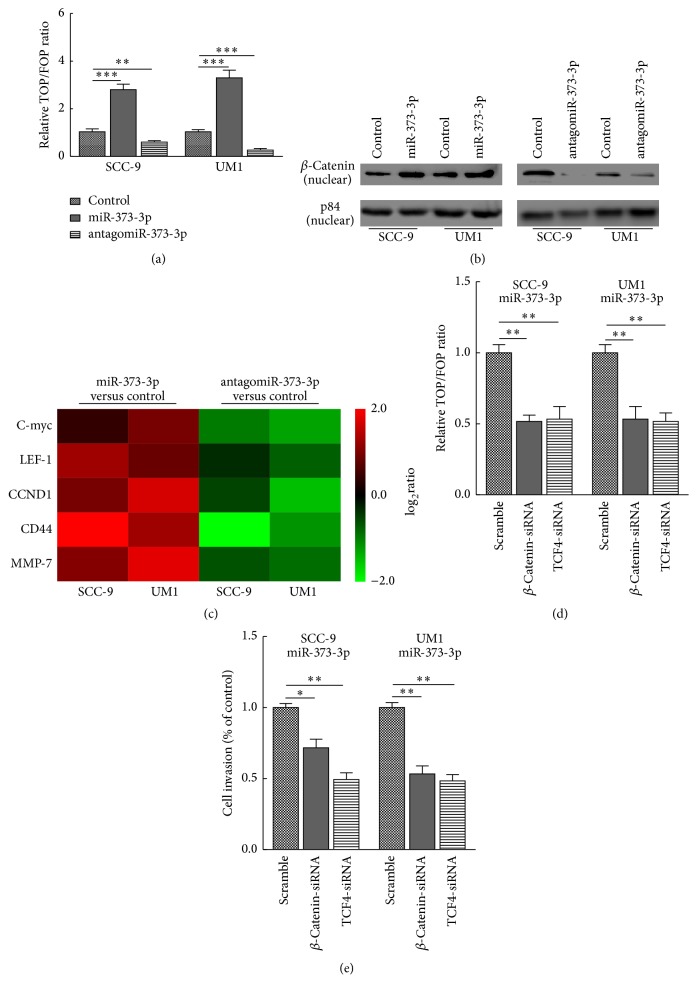
miR-373-3p activates the Wnt/*β*-catenin signaling pathway. (a) TOP/FOP luciferase reporter assays in SCC-9 and UM1 cells transfected with miR-NC (50 nM), miR-373-3p (50 nM), or antagomiR-373-3p (50 nM). (b) Western blotting of nuclear *β*-catenin expression. The nuclear protein p84 was used as a nuclear protein marker. (c) Real-time PCR analysis revealed that miR-373-3p regulates the expression levels of multiple Wnt/*β*-catenin downstream genes. The pseudocolors represent the log_2_ transformed intensity scales of expression in miR-373-3p versus control cells or antagomiR-373-3p versus control cells. (d) The stimulatory effect of miR-373-3p on TOP/FOP luciferase reporter activity was impaired by silencing *β*-catenin or TCF4. (e) Matrigel-coated transwell assay indicated that silencing *β*-catenin or TCF4 abrogated the promotion of TSCC cell invasiveness by miR-373-3p. The data are presented as the mean ± s.d. of three independent experiments. ^*∗*^*P* < 0.05, ^*∗∗*^*P* < 0.01, and ^*∗∗∗*^*P* < 0.001.

Wnt/*β*-catenin signaling is frequently activated in TSCC. Since it is one of the most important pathways in maintaining an epithelial cell phenotype and proper cell-cell junctions and tissue homeostasis [8], we sought to understand whether miR-373-3p affected this pathway. As shown in Figure 3(a), we found that miR-373-3p overexpression significantly increased TOP/FOP luciferase reporter activity, whereas miR-373-3p silencing reduced reporter activity. Besides, Western blot analysis showed that miR-373-3p overexpression increased the nuclear accumulation of *β*-catenin, whereas silencing miR-373-3p reduced nuclear*β*-catenin expression (Figure 3(b)). Apart from this, real-time PCR analysis revealed that miR-373-3p overexpression upregulated several Wnt/*β*-catenin downstream effector genes (Figure 3(c)). Collectively, our results indicate that miR-373-3p activates the Wnt/*β*-catenin signaling pathway in TSCC.

We further explored the functional significance of Wnt/*β*-catenin signaling activation in the miR-373-3p-mediated increase of the invasiveness of TSCC cells by silencing either *β*-catenin or TCF4 in miR-373-3p-overexpressing SCC-9 and UM1 cells. As expected, the stimulation by miR-373-3p of TOP/FOP luciferase reporter activity was impaired by silencing *β*-catenin or TCF4 (Figure 3(d)). Moreover, a Matrigel-coated transwell migration assay indicated that silencing *β*-catenin or TCF4 abrogated the promotion of TSCC cell invasiveness by miR-373-3p (Figure 3(e)). Together, these results suggest that Wnt/*β*-catenin pathway activation is essential for miR-373-3p to promote the invasiveness of TSCC.

### 3.4. miR-373-3p Directly Targets the Wnt/*β*-Catenin Signaling Repressor DKK1

**Figure 4:**
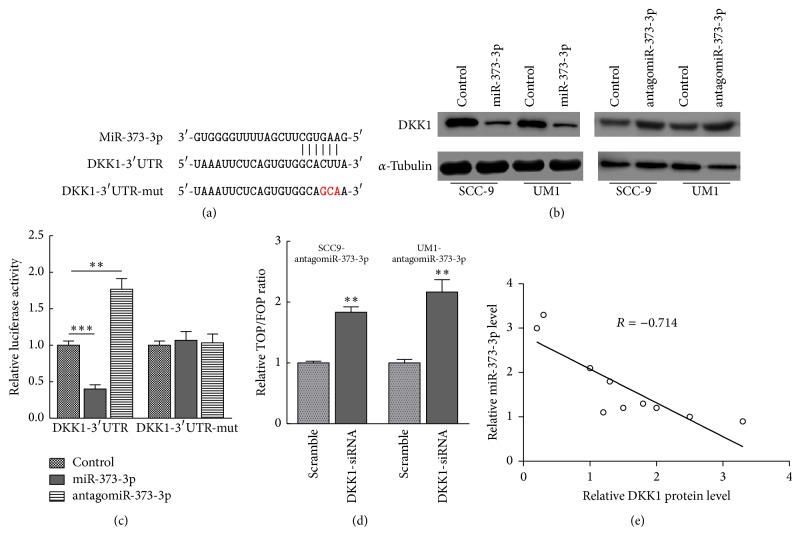
DKK1, a repressor of the Wnt/*β*-catenin signaling pathway, is a direct target of miR-373-3p. (a) The sequence of miR-373-3p matches the 3′-UTR of DKK1 (top). Bottom, mutations of the 3′-UTR of DKK1. (b) DKK1 protein expression was assessed in SCC-9 and UM1 cells transfected with the miR-373-3p mimic or a miR-373-3p inhibitor by Western blot analysis. (c) miR-373-3p inhibited wild-type but not mutated DKK1 3′-UTR luciferase reporter activity. (d) Silencing of DKK1 rescued the antagomiR-373-3p-mediated TOP/FOP luciferase reporter activity suppression. (e) Pearson's correlation scatter plot of the fold change of the levels of miR-373-3p and DKK1 mRNA in 10 recurrent TSCC tissue samples (*R* = −0.714, *P* = 0.009). The data are presented as the mean ± s.d. of three independent experiments. ^*∗∗*^*P* < 0.01; ^*∗∗∗*^*P* < 0.001.

To identify potential targets of miR-373-3p, we used the publicly available algorithms miRanda and TargetScan and identified the Wnt/*β*-catenin signaling repressor DKK1 (Figure 4(a)). Western blot analysis revealed that miR-373-3p overexpression markedly reduced the expression of DKK1 in two TSCC cell lines. In contrast, miR-373-3p inhibition increased DKK1 expression, suggesting that miR-373-3p is a negative regulator of DKK1 (Figure 4(b)). Furthermore, a luciferase assay showed that miR-373-3p overexpression attenuated reporter activity with the 3′-UTR of DKK1, whereas miR-373-3p inhibition elevated this activity (Figure 4(c)). However, ectopic miR-373-3p expression did not repress the reporter activity with a mutant DKK1 3′-UTR (Figure 4(c)). In addition, silencing DKK1 expression potently rescued the TOP/FOP luciferase reporter activity in miR-373-3p-silenced cells (Figure 4(d)), demonstrating that DKK1 is a functional effector of miR-373-3p in regulating Wnt/*β*-catenin signaling activation. To further validate that DKK1 is a direct target of miR-373-3p, we investigated the relationship between miR-373-3p and DKK1 expression in tissues isolated from TSCC patients and found that DKK1 levels are inversely correlated with miR-373-3p expression levels (Figure 4(e)). Taken together, our results suggest that miR-373-3p activates Wnt/*β*-catenin signaling by directly targeting DKK1.

## 4. Discussion

As hallmarks of aggressive cancer, invasion and metastasis lead to poor prognosis in patients. Exploring the underlying mechanisms of cell migration and invasion is crucial to better understand cancer metastasis and discover potential therapeutic targets. Recent work suggests that miRNAs are important molecular regulators that can modulate cell migration and invasion [20]. Hence, clarifying the potential regulatory mechanism of miRNAs in tumor development may provide novel diagnostic and therapeutic perspectives for malignancy. In this study, qRT-PCR validation results revealed that miR-373-3p expression was enhanced in TSCC tissues compared with the paired adjacent normal tissues. Apart from this, increased expression of miR-373-3p in TSCC tissues was significantly associated with a more aggressive TSCC phenotype.

Several researches have suggested that miR-373-3p has a role in multiple cellular processes including proliferation, differentiation, senescence, and apoptosis. These studies also suggested that deregulation is a characteristic of human cancer [16]. miR-373-3p is upregulated in diverse cancers, including lung cancer [21], breast cancer [22], gastric cancer [23], and testicular germ cell tumors (TGCTs) [24]. Voorhoeve et al. [24] showed that expression of miR-373-3p was increased in TGCT tissues and it promoted TGCT cell migration and invasion in vitro. Huang et al. [22] clarified that miR-373-3p enhances breast cancer cell progression through the posttranscriptional regulation of CD44. miR-373 and miR-520c can also promote migration in human fibrosarcoma HT1080 cells by directly targeting mTOR and SIRT1, which are negative regulators of MMP9 expression [25]. In addition to miR-373-3p acting as a potent protooncogenic miRNA through its ability to epigenetically disrupt the biology of the cell, it also functions as a suppressor of cell migration and invasion [16, 26, 27]. Nakata et al. [26] demonstrated that miR-373-3p is downregulated in pancreatic cancer and inhibits cancer cell invasion by increasing the expression of E-cadherin. Zhang et al. [27] identified miR-373-3p as a tumor suppressor that directly targets the Rab22a oncogene in ovarian cancer. In different circumstances and different subcellular distributions, miR-373-3p exhibits complex dysregulation; therefore, miR-373-3p expression may be vastly different during the progression of different tumors. In TSCC, we found that miR-373-3p acted as a potent metastasis-promoting factor. In our studies, miR-373-3p overexpression promoted EMT and invasion. More importantly, miR-373-3p inhibition markedly weakened EMT and the invasive capacity of SCC-9 and UM1 cells. Similar to the above researches, our study clarifies functional role of miR-373-3p as it relates to TSCC progression and metastatic processes.

Several signaling pathways, including Wnt/*β*-catenin, notch, and PI3K/Akt, are aberrantly activated and function in the progression of TSCC [4, 7, 8]. Among these pathways, how Wnt/*β*-catenin signaling functions in TSCC development and progression has been deeply explored [7, 8]. Two critical downstream effectors of the Wnt/*β*-catenin signaling pathway, TCF4 and its coactivator *β*-catenin, are considered to be potent oncogenes in tumor progression [28]. Notably, the expression level and nuclear localization of *β*-catenin are often abnormal in TSCC, which suggests a constitutive activation of Wnt/*β*-catenin signaling [7, 9, 29]. However, how cancer cells evade negative regulation of Wnt/*β*-catenin signaling and establish constitutive *β*-catenin/TCF activation remains unclear. Most importantly, our results established DKK1, a negative regulator of Wnt signaling, as a direct functional effector of miR-373-3p in TSCC, which is in agreement with many studies showing that DKK1 is downregulated in various cancers [30–32]. So far, how DKK1 functions and regulates in TSCC remains unclear. Consistent with previous reports that DKK1 might be implicated in tumor development and progression, our study showed that DKK1 expression was low in primary TSCC tissues and was inversely correlated with miR-373-3p levels. The downregulation of DKK1 by miR-373-3p is likely to contribute to tumor progression in TSCC. DKK1 regulation by miR-373-3p was also detected in TSCC cell lines by Western blotting and a luciferase reporter assay. Our mechanistic and functional data permit us to better appreciate the functional role of DKK1 in TSCC, a situation in which DKK1 negatively regulates Wnt signaling. Our results also indicated that *β*-catenin or TCF4 knockdown suppressed TSCC cell invasion, which phenocopied the effects of miR-373-3p silencing in vitro. These data prove that Wnt signaling plays a role in cell invasion in TSCC and that DKK1 is the direct target site of miR-373-3p.

We have shown that miR-373-3p is a potent protooncogenic factor in TSCC. miR-373-3p upregulation promotes TSCC invasion by downregulating DKK1, causing Wnt signaling activation, thus inducing EMT. As far as we know, our research has proven to be innovative in demonstrating that the miR-373-3p/DKK1/Wnt signaling axis regulates the invasion of TSCC cells. These findings provide a more profound understanding of the development and progression of TSCC and may have important implications for future therapy against TSCC.
